# Does growing up with a physician influence the ethics of medical students’ relationships with the pharmaceutical industry? The cases of the US and Poland

**DOI:** 10.1186/s12910-017-0208-7

**Published:** 2017-08-10

**Authors:** Marta Makowska

**Affiliations:** Warsaw University of Life Sciences, Faculty of Social Sciences, Ul. Nowoursynowska 166, 02-787 Warsaw, Poland

**Keywords:** Medical business ethics, Pharmaceutical marketing, Medical students, Physicians, Poland, US

## Abstract

**Background:**

Medical schools have a major impact on future doctors’ ethics and their attitudes towards cooperation with the pharmaceutical industry. From childhood, medical students who are related to a physician are exposed to the characteristics of a medical career and learn its professional ethics not only in school but also in the family setting. The present paper sought to answer the research question: ‘How does growing up with a physician influence medical students' perceptions of conflicts of interest in their relationships with industry?’

**Methods:**

An anonymous questionnaire was completed by 451 medical students from four Philadelphia medical schools and 554 medical students from Warsaw Medical University during 2013. Medical schools in these two cities were chosen because they are both university cities with similar population sizes. Students who had and who did not have a family member working as a physician were compared using chi-square analysis. Data were analysed for each country separately.

**Results:**

For both the US and Poland, there were statistically significant differences (*p* < .05) between medical students with a physician as a family member and other students with respect to views regarding relationship with the pharmaceutical industry. In both groups, this difference occurred for three important dimensions: students’ relationship with the pharmaceutical industry; students’ views on physicians’ rights to cooperate with the pharmaceutical industry; trust in the pharmaceutical industry. In the US, students related to a doctor were characterized by more restrictive opinions on all three dimensions than other students (e.g., 27.8% of the former students vs. 31.4% of the latter students thought doctors had unrestricted rights to cooperate with the industry). However, the contrary was observed in Poland: students with a physician in the family had less strict views than their colleagues (e.g., 56.8% of the former vs. 39.7% of the latter thought that doctors should have unrestricted rights of cooperation).

**Conclusions:**

In Poland, a former communist country, physicians transmit a more liberal approach towards collaboration with the pharmaceutical industry to their student relatives than those in the US.

## Background

Ethical issues concerning medical students’ relationships with the pharmaceutical industry have been increasingly discussed in the United States of America since research showed their extent [1, 2]. In 2008, the Association of American Medical Colleges introduced guidelines governing this relationship [3]. This was followed by further guidelines issued by the National Academy of Medicine (formerly the Institute of Medicine) [4]. The principles established in these documents say: medical students and university staff should not accept gifts such as pens and food from the industry [3, 4]; either distribution of drug samples should be centrally managed by universities [3] or drugs should only be accepted for poorer patients [4]; relationships between physicians, students and the industry should be transparent [3]; pharmaceutical sales representatives (PSRs) should only meet with physicians only in nonpatient care areas [3, 4]; medical students should only converse with PSRs if they are accompanied by an experienced physician [3]; scholarships and other educational funds from the industry should be centrally administered by academic centers [3]. Also, in 2012 the American Medical Student Association published guidelines about preparing students to interact with business [5].

All of these guidelines have influenced conflict of interest policies in US medical schools. The Pharmfree Scorecard shows that academic institutions have started to create stricter standards for cooperation [6]. Currently, some institutions ban PSRs from entering campuses.

In Poland, the problem of the pharmaceutical industry forming relationships with students has not even been recognized. Polish medical students have frequent contact with PSRs, universities do not have conflict of interest polices, and there is a lack of training in educational programs to defend against the influence of pharmaceutical companies [7].

Medical schools have a great impact on physician socialization and have to support the moral development of students. In recent years the need for a medical business ethics curriculum has been emphasized in the US [3–5]. This curriculum should consider conflicts of interest, administrative fraud and abuse, and the functioning and structure of reimbursement systems [8].

Medical student cooperation with the pharmaceutical industry, such as attending industry-sponsored conferences, meeting PSRs, accepting gifts, etc., is dangerous for many reasons. Students are easily manipulated by pharmaceutical sales representatives because of their limited medical experience and knowledge [9]. This relationship puts medicine in a bad light as the pharmaceutical industry has been involved in a number of scandals in recent years and the relationship can undermine patient trust. Students become used to pharmaceutical promotions and privileges, and when they become physicians such bonds are hard to break. These ties can influence their occupational objectivity. Research shows that physicians who receive gifts from a company prescribe its products more frequently [10, 11]. Conflicts of interest can lead physicians to prioritize their own profits over patient safety and health [8, 12].

Medical students’ attitudes towards the pharmaceutical industry are formed at the start of their medical education [13]. Medical schools not only provide knowledge through the formal curriculum, but also provide models of physician behaviour to be observed and imitated. Learning through observation of models constitutes informal curriculum, and in addition to this hidden curriculum (university’s institutional practices and culture) is also influential [4]. Professional socialization in medicine transmits to students the norms of interpersonal interaction among superiors and subordinates, group identification and solidarity [14].

In the 1960s, US research found that many physicians’ children also become doctors, children often wanting to follow in their parents’ footsteps [15]. It can be assumed that children with a physician parent are better prepared than others for the role of physician because their observation of the profession begins much earlier than their formal medical education. It may be easier for them to adapt to the requirements of a medical school because they have unconsciously assimilated some of the elements of ‘being a doctor’ from home [16]. The family is the most important mechanism in transmitting social values [17], values of physician parents being transmitted to their medical student offspring. For these reasons the research question: ‘How does growing up with a physician influence medical students’ perceptions of conflicts of interest in their relationships with industry?’ was posed. This was part of a larger project aiming to compare the ethical challenges posed by cooperation between medical students and the pharmaceutical industry in the US and Poland.

## Methods

The research was conducted using an anonymous, self-administered questionnaire. The questionnaire contained eight main questions asking medical students about their opinions on different aspects of pharmaceutical marketing. There were also four demographic questions, the most important of which in this article was: Are you related to a physician? The questionnaire and the database are freely available on figshare (DOI: https://doi.org/10.6084/m9.figshare.5067364.v1).

The project aimed to obtain a non-random sample using purposive sampling of at least 100 students from each student year researched in Poland and the US. In each country, students in early years of their courses were excluded since they had the least experience of interacting with the pharmaceutical industry. In Poland, students from the third, fourth, fifth and sixth years of the First and Second Medical Faculty at the Medical University of Warsaw (WUM: the only medical school in Warsaw) were asked to complete the questionnaire during 2013, with 554 questionnaires being completed. In the US, second, third and fourth year medical students in Philadelphia correctly completed the questionnaire. The study was conducted using students at Temple Medical School, Drexel University College of Medicine, Perelman School of Medicine at the University of Pennsylvania, and the Philadelphia College of Osteopathic Medicine. Here, 451 correctly completed questionnaires were gathered. In both countries, the minimum target number of completed questionnaires for each student year was reached or exceeded. The US sample was smaller than the Polish sample because medical school lasts 4 years in the US but 6 years in Poland (Polish medical students do not need to have any ‘pre-med’ university education). Post-stratification weights were applied to ensure that answers from students in each year had the same impact on the results. The sample breakdown after application of the weighting procedure is shown in Table 1.

**Table 1:** Characteristics of the samples

	USA		Poland	
Characteristic	%	N	%	N
Sex				
Female	55.7%	251	60.8%	336
Male	44.3%	200	39.2%	217
Total	100%	451	100%	553
Year of study				
Second	34.1%	154		
Third	31.9%	144	26.2%	145
Fourth	34.1%	154	24.8%	138
Fifth			24.2%	134
Sixth			24.8%	137
Total	100%	452	100%	554
School				
Medical University of Warsaw			100%	554
Temple University School of Medicine	23.2%	105		
Drexel University College of Medicine	29.9%	135		
Perelman School of Medicine at the University of Pennsylvania	18.2%	82		
Philadelphia College of Osteopathic Medicine	28.7%	129		
Total	100%	451	100%	551
Are you related to a physician?				
Yes	29.0%	131	30.9%	170
No	71.0%	320	69.1%	381
Total	100%	451	100%	551

Source: M. Makowska & M. Etyczne. Wyzwania współpracy studentów medycyny z przemysłem farmaceutycznym. Studium porównawcze sytuacji w Polsce i USA. Warszawa: Wydawnictwo SGGW; 2016, p. 189

In the US, the weighting design also included respondents’ school of attendance because there were different response rates across different universities. The sample weights are presented in Table 2.

**Table 2:** Sample weights

	University				
Year of study	Temple University	Drexel University	Perelman School	PCOM	Medical University of Warsaw
Second	0.91	0.80	1.37	1.14	
Third	1.45	0.90	0.85	1.38	1.42
Fourth	1.80	0.70	0.68	1.17	1.33
Fifth					1.28
Sixth					0.56

While the Polish part of the study was done as planned using an in-class method of research, in the US, due to the organization of classes, students completed an online survey. The possibility of a mode effect therefore arose, and consideration was given to repeating the Polish part of the research in Poland with an on-line sample, but this was ultimately rejected. Potential drawbacks included the possibility of a very small response rate, sensitization of some respondents to the topic, and the temporal inconsistency of responses across the 1 year time gap that would be likely to have occurred. Moreover studies of the mode effect comparing in-class and on-line surveys [18–20] suggest that this effect would have been rather small.

It is worth emphasizing that in both countries student participation was voluntary, and the data collection modality influenced readiness to participate. For in-class collection most students (around 80%) completed the questionnaire (more doing this when it was done at the start of a lesson than at the end). Data collection ceased when the number of students for each year exceeded 100. In total 554 valid questionnaires was gathered from the respondents (in WUM there was 1841 students meeting the criteria to take part in the survey, not all of them were asked to complete the questionnaire).

In the US, the request to complete the survey was sent to all 2819 students meeting the criteria. In each medical school an email message was sent by a teacher from the school, so the participation requested would not be treated as junk email. A problem with this method was that it was difficult to assess how many students read or even opened the message. Only 16% (451) of students who met the criteria completed the questionnaire. One of the consistently highlighted disadvantages of internet surveys compared to in-class data collection methods is the lower percentage of returns [19, 20].

Although data from neither of the countries can be said to have been collected from a representative group, the samples provided a valuable source of information about students’ opinions and their knowledge of pharmaceutical marketing. The study was limited in that funding constraints precluded more widely ranging data collection in the two countries involved, but Philadelphia and Warsaw are both university cities with similar population sizes, and to this extent the two cities should provide a good comparison between Poland and the US (and for reasons of brevity, in the text below the terms Polish and US are used to refer to students participating in the present study).

Descriptive statistics, and relative and absolute frequencies were calculated separately for both countries. Inferential tests mostly consisted of Pearson’s chi-square tests to analyze differences between the countries, but Mann-Whitney tests and Kruskal-Wallis tests were also used (for more detail see [7]). The data set was analyzed using IBM SPSS Statistics 22. This article will describe only a small, but the most interesting and unexpected, part of the research.

### The orgin of the indexes

In the Results section below, statistically significant differences (*p* < .05) between medical students with a physician as a family member and other students are described with respect to three indexes: a reciprocity index, an index of physicians’ rights and a trust index. These indexes resulted from principal components analyses performed separately for each country. Principal components analysis (PCA) was done separately for Question 1 where there were eight statements concerning students’ opinions about the pharmaceutical marketing aimed at them, and for Question 6 where there were 12 statements concerning students’ opinions about the pharmaceutical marketing aimed at practising physicians. Students could strongly agree, rather agree, rather disagree and strongly disagree with each statement. The PCAs were performed towards the end of using the sets of statements to build internally consistent indexes to describe attitudes occurring in the study population.

### Students’ opinions of their own cooperation with the pharmaceutical industry

For the Polish students’ questionnaires, PCA with varimax rotation was used to isolate components. During PCA two statements were excluded from analysis. First, the statement ‘Sudies are preparing me well for future cooperation with pharmaceutical companies’ representatives’ was excluded because its Kaiser-Meyer-Olkin (KMO) measure of sampling adequacy (in the diagonal of the anti-image matrix) had a value below .50, indicating that it had little variance in common with other statements in the analysis. Additionally, after much consideration, the sentence ‘If I got a textbook for free, I would not mind if the textbook had a pharmaceutical company logo on every page’ was removed because it interfered with the analysis.

In a final run, six statements were included in the analysis. Kaiser’s criterion indicated two components. For the analysis as a whole the KMO measure of sampling adequacy attained a value of .728, which indicated that responses to statements had enough variance in common to permit an adequate PCA. A significant Bartlett’s test of sphericity (*p* < .001) also indicated that an identity matrix did not exist, indicating that there was no problem with a lack of correlational relationships between responses to statements (see Table 3 for rotated loadings and communalities).

**Table 3:** PCA of statements concerning Polish students’ cooperation with the pharmaceutical industry

Statement (4-point Likert Scale)	Component 1	Component 2	*h* ^2^
During their studies medical students should have contact with pharmaceutical industry representatives.	.152	**.852**	.749
Lectures, seminars, and presentations organized by pharmaceutical companies should be a part of the education of medical students.	.154	**.691**	.501
Medical students should have the right to perform paid work for pharmaceutical companies.	**.428**	.336	.296
Medical students should have the right to take gifts of modest value which are needed in their education (e.g., pens, notebooks) from pharmaceutical companies.	**.684**	.235	.523
Medical students should have the right to take expensive gifts from pharmaceutical companies if they are needed in their education (e.g., branded electronic stethoscopes).	**.949**	.158	.925
Medical students should have the right to take gifts from pharmaceutical companies in any form in which companies are prone to give them (e.g., good quality wine).	**.689**	.079	.480
Eigenvalue	2.07	1.40	SUM
Variance explained	34.5%	23.4%	57.9%

Source: M Makowska & M. Etyczne. Wyzwania współpracy studentów medycyny z przemysłem farmaceutycznym. Studium porównawcze sytuacji w Polsce i USA. Warszawa: Wydawnictwo SGGW; 2016, p. 196. Loadings in bold indicate the highest component loading for each item (these being the most salient loadings for interpretation of each component)

Again using PCA with varimax rotation, two statements were also excluded from the PCA involving US students’ responses, this time on the grounds that the statements had communalities below .25. These statements were: ‘Studies are preparing me well for future cooperation with pharmaceutical companies’ representatives’ and ‘Medical students should have the right to perform paid work for pharmaceutical companies’. Using Kaiser’s criterion, two components were again identified. Both the overall KMO measure of sampling adequacy (.785) and Bartlett’s test of sphericity (*p* < .001) were acceptable. The amount of variance explained was slightly larger (64%) than for the Polish sample (57.9%). Table 4 presents rotated loadings and communalities for the analysis.

**Table 4:** PCA of statements concerning US students’ cooperation with the pharmaceutical industry

Statement (4-point Likert Scale)	Component 1	Component 2	*h* ^2^
During their studies medical students should have contact with pharmaceutical industry representatives.	.220	**.794**	.679
Lectures, seminars, and presentations organized by pharmaceutical companies should be a part of the education of medical students.	.238	**.788**	.678
If I got a textbook for free, I would not mind if the textbook had a pharmaceutical company logo on every page.	**.491**	.325	.347
Medical students should have the right to take gifts of modest value which are needed in their education (e.g., pens, notebooks) from pharmaceutical companies.	**.694**	.359	.610
Medical students should have the right to take expensive gifts from pharmaceutical companies if they are needed in their education (e.g., branded electronic stethoscopes).	**.869**	.215	.801
Medical students should have the right to take gifts from pharmaceutical companies in any form in which companies are prone to give them (e.g., good quality wine).	**.839**	.148	.726
Eigenvalue	2.28	1.55	SUM
Variance explained	51.0%	13.0%	64.0%

Source: M Makowska & M. Etyczne. Wyzwania współpracy studentów medycyny z przemysłem farmaceutycznym. Studium porównawcze sytuacji w Polsce i USA. Warszawa: Wydawnictwo SGGW; 2016, p. 202. Loadings in bold indicate the highest component loading for each item (these being the most salient loadings for interpretation of each component)

Despite some differences in the composition of Component 1 across the two countries because of deletion of certain statements mentioned above, the first component was given the same interpretation for Polish and US students (see Tables 3 and 4): students’ opinions about receiving gifts from the pharmaceutical industry. This component was used to create the reciprocity index described below. Again being similar for the two countries, the second component was interpreted as tapping students’ opinions about the participation of pharmaceutical companies in their education. This component was also use to create an index, but this is not described in the Results section because there were no statistically significant differences in this index for students having and not having a physician in their family.

### Students’ opinions of physicians’ cooperation with the pharmaceutical industry

The same techniques as used above (PCA with varimax rotation) was used to analyze students’ opinions about physician’s cooperation with the pharmaceutical industry. For both countries, Kaiser’s criterion identified three components. During interpretation of components for both countries the statement ‘Gifts to physicians from pharmaceutical companies are also beneficial for patients’ was removed because it made interpretation of Component 1 more difficult. For the Polish analysis, the overall KMO statistic had a value of .792, and Bartlett’s test of sphericity was significant (*p* < .001). For the US data, the KMO statistic had a value of .837, and again Bartlett’s test of sphericity was significant (*p* < .001). Component loadings and communalities for the two analyses are given in Tables 5 and 6.

**Table 5:** PCA of statements concerning Polish students’ opinions about physicians’ cooperation with the pharmaceutical industry

Statement (4-point Likert Scale)	Component 1	Component 2	Component 3	*h* ^2^
Physicians should have the right to take expensive gifts from pharmaceutical companies if they are needed in their practice (e.g., branded stethoscopes).	**.853**	.133	−.104	.757
Physicians should have the right to get support from pharmaceutical companies to attend conferences, training events, etc.	**.747**	.256	−.077	.629
Physicians should have the right to take gifts from pharmaceutical companies in any form in which companies are likely to give them (e.g., good quality wine).	**.736**	−.019	.099	.552
Physicians should have the right to take gifts of modest value which are needed in their practice (e.g., pens, notebooks) from pharmaceutical companies.	**.732**	.244	−.145	.617
Physicians should have the right to perform paid work for pharmaceutical companies (e.g., completing questionnaires or examining patients).	**.646**	.214	.041	.465
Pharmaceutical sales representatives perform an important educational function for physicians.	.102	**.818**	.068	.685
Physicians should meet with medical representatives during working hours because they are getting information about the drugs necessary in their work.	.259	**.736**	−.075	.615
Information about drugs coming from pharmaceutical companies is credible.	.160	**.614**	−.143	.422
Gifts from pharmaceutical companies are rewards for prescribing their medicines.	−.201	.065	**.820**	.716
Gifts to physicians from pharmaceutical companies influence physicians’ prescribing habits.	−.083	−.066	**.817**	.679
Drug samples are also gifts from a pharmaceutical company.	.110	−.085	**.401**	.180
Eigenvalue	2.94	1.79	1.58	SUM
Variance explained	26.7%	16.3%	14.4%	57.4%

Source: M Makowska & M. Etyczne. Wyzwania współpracy studentów medycyny z przemysłem farmaceutycznym. Studium porównawcze sytuacji w Polsce i USA. Warszawa: Wydawnictwo SGGW; 2016, p. 252. Loadings in bold indicate the highest component loading for each item (these being the most salient loadings for interpretation of each component)

**Table 6:** PCA of statements concerning US students’ opinions about physicians’ cooperation with the pharmaceutical industry

Statement (4-point Likert Scale)	Component 1	Component 2	Component 3	*h* ^2^
Physicians should have the right to take expensive gifts from pharmaceutical companies if they are needed in their practice (e.g., branded electronic stethoscopes).	**.873**	.133	−.093	.789
Physicians should have the right to take gifts from pharmaceutical companies in any form in which companies are likely to give them (e.g., good quality wine).	**.834**	.068	−.056	.704
Physicians should have the right to take gifts of modest value which are needed in their practice (e.g., pens, notebooks) from pharmaceutical companies.	**.695**	.263	−.204	0,594
Physicians should have the right to get support from pharmaceutical companies to attend conferences, training events, etc.	**.666**	.309	−.073	.544
Physicians should have the right to perform paid work for pharmaceutical companies (e.g., completing questionnaires or examining patients).	**.608**	.160	.053	.398
Pharmaceutical sales representatives perform an important educational function for physicians.	.218	**.810**	.005	.704
Information about drugs coming from pharmaceutical companies is credible.	.082	**.759**	−.090	.591
Physicians should meet with medical representatives during working hours because they are getting information about the drugs necessary in their work.	.299	**.674**	−.137	.562
Gifts from pharmaceutical companies are rewards for prescribing their medicines.	−.149	−.041	**.742**	.574
Gifts to physicians from pharmaceutical companies influence physicians’ prescribing habits.	−.412	.001	**.671**	.620
Drug samples are also gifts from a pharmaceutical company.	.210	−.145	**.580**	.401
Eigenvalue	3.13	1.92	1.43	SUM
Variance explained	28.5%	17.4%	13.0%	58.9%

Source: M Makowska & M. Etyczne. Wyzwania współpracy studentów medycyny z przemysłem farmaceutycznym. Studium porównawcze sytuacji w Polsce i USA. Warszawa: Wydawnictwo SGGW; 2016, p. 253–254. Loadings in bold indicate the highest component loading for each item (these being the most salient loadings for interpretation of each component)

Exactly, the same interpretations of the three components applied to Polish and US students. The first component was interpreted as tapping opinions about the rights of physicians to cooperate with the pharmaceutical industry, and was used to create the index of physicians’ rights described below. The second component was interpreted as being concerned with trusting information from pharmaceutical companies and was used to create the trust index, also described below. The last, third, component was named ‘awareness of the impact of gifts from pharmaceutical companies on physicians’. This was also used to form an index, but there were no significant differences on this index between students having and not having a physician in their family, so it is not described below.

## Results

In both countries, students who had a doctor as a family member were significantly differentiated from other medical students. Many statements indicated their varying approach to cooperation with the pharmaceutical industry. In the US, those with such a relative were characterized by more restrictive attitudes than their colleagues. Surprisingly, in Poland, students who were related to a doctor had less restrictive views on cooperation with pharmaceutical companies than other students. Differences between students were statistically significant on three important dimensions: students’ opinions about cooperation with the pharmaceutical industry, students’ view on physicians’ rights to cooperate with the pharmaceutical industry and trust in the pharmaceutical industry.

### Students’ cooperation with the pharmaceutical industry

A reciprocity index was created from four statements. Three of these were the same in both countries: (1)‘Medical students should have the right to take gifts of modest value and needed in education (e.g., pen, notebook) from pharmaceutical companies.’; (2) ‘Medical students should have the right to take expensive gifts from pharmaceutical companies if they are needed in their education (e.g., brand electronic stethoscope).’ (3) ‘Medical students should have the right to take gifts from pharmaceutical companies in any form in which companies are willing to give them (e.g., good quality wine).’ Only one was different.^1^ (4) If I got a textbook for free, I would not mind the textbook having a pharmaceutical company logo on every page. (US)/Medical students should have the right to perform paid work for pharmaceutical companies. (Poland). All statements were intended to ascertain students’ beliefs about different gifts and possibilities of cooperation with the industry which might result in a perceived obligation to repay industry members because of the concept of reciprocity. For both the US and Poland Cronbach’s alpha exceeded .80, indicating that both indexes had good internal consistency.

A reciprocity index was calculated for each student. Respondents received points for their answer to each statement (1p – strongly agree, 2p – somewhat agree, 3p - somewhat disagree and 4p – strongly disagree). Finally, four groups of students with different attitudes toward cooperation that may lead to a perceived need for reciprocity were distinguished: very good collaborators (4–7 p), good collaborators (8–10 p), weak opponents (11–13 p), strong opponents (14–16 p).

In the US, students with a physician in their family were most frequently strong opponents of cooperation with the industry (34.6%), but the contrast group were most commonly good co-operators (33.6%). In Poland, students with a physician in the family were most commonly very good collaborators (34.7%) while the contrast group were most frequently good collaborators (35.4%). This suggests that having a physician in the family makes Polish student more willing to cooperate with the pharmaceutical industry. Additionally, in the US most respondents were good collaborators (29.5%) and weak opponents (27.7%), while in Poland most students were good (32.8%) or very good collaborators (27.0%). Inferential statistics were computed for each country separately and for both countries Pearson’s chi-square tests for the association between having / not having a physician in the family and reciprocity index grouping were statistically significant (Table 7).

**Table 7:** Reciprocity indices for Polish and US students having and not having a family member working as a physician

Reciprocity index	US			POLAND		
	Students who have a physician as a family member	Students who do not have a physician as a family member	All students	Students who have a physician as a family member	Students who do not have a physician as a family member	All students
Very good collaborator	17.3%	19.5%	18.8%	34.7%	23.5%	27.0%
Good collaborator	19.5%	33.6%	29.5%	26.9%	35.4%	32.8%
Weak opponent	28.6%	27.4%	27.7%	25.1%	27.7%	27.0%
Strong opponent	34.6%	19.5%	23.9%	13.2%	13.2%	13.2%
Sum	100% (*n* = 133)	100% (*n* = 318)	100% (*N* = 451)	100% (*n* = 167)	100% (*n* = 378)	100% (*N* = 545)

US: *X* ^2^ (3, *N* = 451) = 15.5, *p* = .01

PL: *X* ^2^ (3, *N* = 545) = 8.2, *p* < .05

### Students’ views on physicians’ rights to cooperate with the pharmaceutical industry

The index of physicians’ rights was based on the same five items for both countries: (1) ‘Physicians should have the right to take gifts of a modest value and needed in the practice (e.g., pen, notebook) from pharmaceutical companies.’; (2) ‘Physicians should have the right to take expensive gifts from pharmaceutical companies if they are needed in their practice (e.g., brand/ electronic stethoscope).’; (3) ‘Physicians should have the right to take gifts from pharmaceutical companies in any form in which companies are likely to give them (e.g., good quality wine).’; (4) ‘Physicians should have the right to get support from pharmaceutical company to attend conferences, trainings etc.’; (5) ‘Physicians should have the right to perform paid work for pharmaceutical companies (e.g., filling the questionnaires or examine patients).’ All of these statements considered students’ views about practising doctors’ rights to cooperate with the pharmaceutical industry. For both Poland and the US Cronbach’s alphas above *.*80 again indicated good internal *consistency of the physicians’ rights index.*

In this index students received 1 point for all ‘strongly agree’ and ‘somewhat agree’ answers, and no points for somewhat disagree and strongly disagree. Respondents were divided into three groups: those attributing no rights or very restricted rights to physicians (0-1p); those attributing some rights to physicians (2–3 p); those attributing full or almost full rights (4-5p) to physicians in their cooperation with the pharmaceutical industry.

The majority of the US students (42.4%) attributed some rights to physicians, but for those with a doctor in the family the largest number of respondents (38.3%) were assigned to the group attributing no or almost no rights to physicians. In Poland most respondents (44.9%) attributed physicians full and almost full rights (as many as 56.8% of students with a family member working as a physician and 39.7% of the contrast group). Chi-square tests, conducted separately for Polish and US students, for the association between having / not having a physician in the family and grouping on the doctor’s rights index revealed statistically significant associations.

Note that two of the rights (numbers 2 and 3) violate Polish pharmaceutical law [21] and all of them are questionable under the professional ethics code. In Philadelphia the law is less severe, and all of the rights are in accordance with the law, but despite this students took a stricter view as shown by the percentages in the all students column for US students in Table 8).

**Table 8:** Doctors’ rights indices for US and Polish students having and not having a family member working as a physician

Doctors’ rights index	US			POLAND		
	Students who have a physician as a family member	Students who do not have a physician as a family member	All students	Students who have a physician as a family member	Students who do not have a physician as a family member	All students
Full and almost full rights	27.8%	31.4%	30.4%	56.8%	39.7%	44.9%
Some rights	33.8%	45.9%	42.4%	32.0%	43.9%	40.2%
Almost no or no rights	38.3%	22.6%	27.3%	11.2%	16.4%	14.9%
Sum	100% (*n* = 133)	100% (*n* = 318)	100% (*N* = 451)	100% (*n* = 169)	100% (*n* = 383)	100% (*N* = 552)

US: *X* ^2^ (2, *N* = 451) = 12.1, *p* < .01

PL: *X* ^2^ (2, *N* = 552) = 13.9, *p* = .001

### Trust in the pharmaceutical industry

For both countries a trust index was constructed based on respondents’ agreement with the following three sentences: (1) ‘Pharmaceutical sales representatives are performing an important educational function for physicians.’; (2) ‘Information about drugs coming from pharmaceutical companies is credible.’; (3) ‘Physicians should meet with medical representatives during working hours, because they are getting information about the drugs necessary in their work.’ Cronbach’s alpha was above .69 for US, and above .63 for Polish samples, showing moderate internal consistency [22].

Students received one point for answers of strongly agree and somewhat agree, and no points for somewhat disagree and strongly disagree, with students then being placed in four groups: those with no trust at all in information from the industry (0 points), little trust (1 point), reasonable trust (2 points) and high trust (3 points).

US students were more trustful than Polish students: only 27.0% of them had no trust and 29.4% had little trust in information from the pharmaceutical industry. In Poland, 39.6% had no trust and 36.1% had little trust. A chi-square test showed a statistically significant difference between the countries (*p* < .001). In the US, students from a family with a physician were more distrustful than others of companies’ information, while in Poland such students were less doubtful than their colleagues. Chi-square analyses for US showed statistically significant associations (*p* < .01) between having / not having a physician in the family. Grouping on the trust index differences in observed and expected frequencies being in the aforementioned (contrary) directions for the two different countries (Table 9).

**Table 9:** Trust indices for US and Polish students having and not having a family member working as a physician

Trust index	US			POLAND		
	Students who have a physician as a family member	Students who do not have a physician as a family member	All students	Students who have a physician as a family member	Students who do not have a physician as a family member	All students
No trust	37.6%	22.6%	27.0%	35.5%	41.4%	39.6%
Little trust	28.6%	29.8%	29.4%	37.9%	35.3%	36.1%
Reasonable trust	18.0%	26.3%	23.9%	13.6%	16.8%	15.8%
High trust	15.8%	21.3%	19.7%	13.0%	6.5%	8.5%
Sum	100% (*n* = 133)	100% (*n* = 318)	100% (*N* = 451)	100% (*n* = 169)	100% (*n* = 382)	100% (*N* = 551)

US: *X* ^2^ (3, *N* = 451) = 12.0, *p* < .01

PL: *X* ^2^ (3, *N* = 551) = 7.2, *p* = .053

## Discussion

Usually, students’ attitudes toward pharmaceutical marketing are similar to the conflict of interest policy of their medical school. Students from schools with rigorous policies have more critical views of the industry than those from other schools [1, 23]. If their school has an impact on a student it is not surprising that a physician family member also affects them. The most interesting observation of the present study is the dissimilarity in this influence between the US and Poland. The results suggest that in Poland most physicians in a family pass on a conviction to their student offspring that cooperation with the pharmaceutical industry is beneficial, that they should be cooperative, attribute doctors’ the right to cooperate with businesses, and treat information from the industry as not being excessively biased. In the US, doctors in families appear to influence the views of students in the opposite direction: to be more rigorous in their attitudes towards their relationship with the industry. In explaining this difference it is important to examine the history of drug promotion, pharmaceutical marketing law and the cultural background of the two countries.

In the US the first chapters of codes of conduct dealing with how to behave in relationships with industry were created towards the end of the twentieth century (e.g., the AMA’s Code of Medical Ethics, 1990) and a discussion started about the ethics of cooperation. Present US federal law is not very strict about pharmaceutical marketing and does not limit gift-giving, provision of drug samples, or physicians’ meetings with PSRs. This said, *The Physician Payment Sunshine Act (Open Payments)*, which came into force in 2013, slightly tightened the law, enforcing greater transparency in relations between physicians and the pharmaceutical industry. The lack of any strict federal pharmaceutical marketing laws means that self-regulation plays an important role in the US. Industry members take the view that it is better to limit their own actions in accordance with social expectations than to lobby for changes to the law that would be more favorable to them. There are now many codes of conduct concerning this topic, not only industry but also for physicians and medical students. The doctors and medical students can attend scientific meetings to learn how to avoid unwanted ties. There are also organizations such as *No free lunch, Public Citizens,* and the *Pew Prescription Project* aiming to limit physicians’ contact with the industry.

When the first codes of conduct for relationships with industry were created in the US, Polish international pharmaceutical companies had just started to come into the market. Poland is a post-communist country, the system changing in 1989. Any drug advertisement was forbidden until 1993 [24] when over the counter drugs promotion became legal. The lack of experience, inadequacy of pharmaceutical advertisement law, absence of codes of conduct and the acceptance of corrupt behaviours, which are an echo of the former social-political system, makes it easy for Polish doctors to become too close to the pharmaceutical industry. Although the situation improved in 2007 and 2008 when new regulations came into effect. Polish law is now even stricter than European Commission Directives 2001/83/EC and 2004/24/EC [for more details see: 25]. The regulations stipulate that PSRs are not allowed to meet physicians during their working hours, physicians can only accept low value gifts (worth no more than 100 PLN or 33 USD) and that such gifts must have a connection to medical practice. Rx sampling of drugs is unavailable to medical students, nurses, other medical staff and patients, and there is no direct-to-customer advertising in Poland. Polish pharmaceutical companies and physicians’ organizations develop voluntary guidelines, but their codes in crucial areas are no stricter than the law.

In the US, the voluntary guidelines in codes of conduct are taken much more seriously among members of medical society than they are in Poland, where the provisions of codes are often undermined by the view that the law is what really matters and that physicians and PSRs should follow this rather than voluntary guidelines [22]. This said, in Poland it is seen as reasonable to acquiescence in disobeying the law if it is seen as unclear or regarded as ‘stupid’ [7].

There is also a lack of non-governmental organizations aiming to limit Polish doctors’ contacts with the industry. A great number of Polish doctors still see cooperation with the pharmaceutical industry as their privilege and ignore the law during their contact with PSRs [25].

In Poland (the sixth largest pharmaceutical market in Europe), the most important players are the same companies that are active in the US market. They use the same methods to influence physicians and medical students (e.g., gift-giving) in both countries. Unfortunately, there is a large deficiency in polish physicians’ education about conflicts of interest [26] and other issues connected with pharmaceutical marketing. This deficiency applies to both practising Polish physicians and medical students.

Polish and US students were asked what they had currently learned about cooperation with the pharmaceutical industry during their studies. Topics mentioned were: being skeptical of information provided by the industry; methods of finding reliable data about drugs; methods of influence used by PSRs; legal and ethical standards for relationships between physicians and pharmaceutical industry; potential and existing conflicts of interest. All topics were brought up to a statistically significant lesser extent by students in Polish medical schools than those in US medical education. Data also showed that a majority of students from both countries felt unprepared by their schools to cooperate in a correct manner with business (78.7% of US respondents and 87.6% of Polish respondents). This supports the idea that changes in the curricula are needed in both countries [7].

Further, although no Polish medical school has a policy governing the relationships of students and staff with business, the study’s results indicate that there is a need to introduce such policies. Given the lack of such policies in Poland, it is unsurprising that US students showed stricter attitudes toward the pharmaceutical industry than Polish students. They were less willing to cooperate, attributing fewer rights to physicians in cooperating with businesses. Nevertheless, encouragingly, the trust index showed that Polish students were more suspicious than those in the US. This can be explained by the crisis of trust that is apparent in many post-communist countries [27].

The issue arises as to whether the current findings are relevant to countries other than the US and Poland. In the absence of such research one can only speculate that the situation will be similar to that in Poland in countries that are not particularly wealthy and where doctors salaries are less than in richer countries (in Poland the average salary for a doctor is around 17,500 USD a year [28], and in the US it is around 140,000 USD [29]), and in countries where there is no significant self-regulation and a lack of organizations to educate physicians and medical students about relationships with business. In richer countries, and where there are strong guidelines and educational organizations concerned with ethics, the situation is more likely to resemble that in the US. This is an area for further research.

## Conclusions

Although changes in the US medical business ethics curriculum have been proposed [5], both medical schools and practising physicians provide students a better example of how to cooperate with the pharmaceutical industry than in Poland, where changes are definitely needed. First, changes in the curricula of medical schools are needed. During medical ethics classes there is a lack of time or unwillingness to talk about cooperation with the pharmaceutical industry, so students lack basic knowledge about it. Second, schools need to create policies about cooperation with the industry. Third, there is a need for the training of practising physicians about conflicts of interest and methods of manipulation used by the pharmaceutical industry.

## Abbreviations

AMA: American Medical Association
OTC: Over the counter
PSR: Pharmaceutical sales representative

## Footnotes

1 This resulted from Principal Component Analysis (PCA) conducted separately for Polish and US students.
